# Private health care coverage and increased risk of obstetric intervention

**DOI:** 10.1186/1471-2393-14-13

**Published:** 2014-01-13

**Authors:** Jennifer E Lutomski, Michael Murphy, Declan Devane, Sarah Meaney, Richard A Greene

**Affiliations:** 1 National Perinatal Epidemiology Centre, Cork University Maternity Hospital, Wilton, Cork, Ireland; 2 Nijmegen Centre for Evidence Based Practice, Radboud University Medical Centre, Nijmegen, Netherlands; 3 School of Nursing and Midwifery, National University of Ireland, Galway, Ireland

**Keywords:** Caesarean section, Vacuum extraction, Obstetric forceps, Induction of labour, Episiotomy, Pregnancy

## Abstract

**Background:**

When clinically indicated, common obstetric interventions can greatly improve maternal and neonatal outcomes. However, variation in intervention rates suggests that obstetric practice may not be solely driven by case criteria.

**Methods:**

Differences in obstetric intervention rates by private and public status in Ireland were examined using nationally representative hospital discharge data. A retrospective cohort study was performed on childbirth hospitalisations occurring between 2005 and 2010. Multivariate logistic regression analysis with correction for the relative risk was conducted to determine the risk of obstetric intervention (caesarean delivery, operative vaginal delivery, induction of labour or episiotomy) by private or public status while adjusting for obstetric risk factors.

**Results:**

403,642 childbirth hospitalisations were reviewed; approximately one-third of maternities (30.2%) were booked privately. After controlling for relevant obstetric risk factors, women with private coverage were more likely to have an elective caesarean delivery (RR: 1.48; 95% CI: 1.45-1.51), an emergency caesarean delivery (RR: 1.13; 95% CI: 1.12-1.16) and an operative vaginal delivery (RR: 1.25; 95% CI: 1.22-1.27). Compared to women with public coverage who had a vaginal delivery, women with private coverage were 40% more likely to have an episiotomy (RR: 1.40; 95% CI: 1.38-1.43).

**Conclusions:**

Irrespective of obstetric risk factors, women who opted for private maternity care were significantly more likely to have an obstetric intervention. To better understand both clinical and non-clinical dynamics, future studies of examining health care coverage status and obstetric intervention would ideally apply mixed-method techniques.

## Background

When clinically indicated, common obstetric interventions, such as caesarean delivery, operative vaginal delivery (i.e. vacuum or forceps extraction), induction of labour and episiotomy, can greatly improve maternal and neonatal outcomes. However, variation in intervention rates by socioeconomic indicators, such as type of health care coverage, suggests that obstetric practice may not be solely driven by case criteria. Disparities in caesarean delivery rates between women with public versus private health care coverage have been well reported in the United States [1-3], Australia [4,5], China [6], Europe [7-9] and Latin America [10,11]. Differences in incidence rates of operative vaginal delivery, induction of labour and episiotomy have also been observed [4,5,12].

Thus, we explored the association between private maternity coverage and obstetric intervention in Ireland, a small country (~4 million residents) which offers universal maternity benefits to the 75,000 women who deliver each year in its jurisdiction. The current Irish model of maternity care was originally devised under the 1954 Maternity and Infant Care Scheme, which granted women ordinarily resident in Ireland access to free maternity services throughout pregnancy and up to six weeks postpartum [13]. Maternity care is jointly supervised between a general practitioner and a maternity health care provider. Under the public scheme, a woman may be seen by several different obstetricians and/or midwives throughout her pregnancy; low risk, non-operative vaginal deliveries are largely assisted by midwives. For an additional fee, women may opt for private maternity care, and in this case, care is alternated between a general practitioner and a consultant obstetrician, who will attend the delivery. Women may choose the consultant, or if a preference is not stated, a consultant obstetrician will be assigned. There are 20 maternity units across Ireland, of which 19 are public and one is private; private maternity care can be received at any of the public hospitals.

With this backdrop, the aim of our study was to complement current research in this area by comparing rates of obstetric intervention among women with private versus public health care coverage in the Irish maternity system. While allowing for case-mix variation, we examined differences in rates of elective caesarean and emergency delivery, operative vaginal delivery, induction of labour and episiotomy. We hypothesised, based on the previous literature, that obstetric interventions would be higher among women with private coverage.

## Methods

We performed a population-based retrospective cohort study of deliveries occurring in Irish maternity units between January 1, 2005 and December 31, 2010. Data were extracted from the Hospital In-Patient Enquiry (HIPE) database [14], which provides nationally representative morbidity data coded according to the *International Statistical Classification of Diseases and Related Health Problems, Tenth Revision, Australian Modification* (ICD-10-AM). For every hospital discharge, centrally trained personnel input basic demographics and up 20 diagnoses and/or procedures into identical, standardised data entry forms. These forms are subsequently submitted to the Economic and Social Research Institute, which is the national body responsible for data maintenance and dissemination of hospital activity [14]. In adherence with the Institute’s protocol, HIPE data routinely undergo more than 140 validation checks, and since 2001, 18 chart-based audits have been performed [15-17]. The HIPE clinical coder training program and audit procedures have been independently assessed and were found to be accurate and reliable [15-17]. All 19 public maternity units submit data to HIPE. Thus, given the small obstetric volume of the single private maternity unit in the country (<2,000 deliveries per annum) and the low national home birth rate (<1%), the HIPE database represents approximately 97% of all deliveries [18].

Childbirth hospitalisations were identified using the ICD-10-AM outcome of delivery code, Z37, which indicates the number of gestations (e.g. singleton, twin, or higher multiple order birth) and birth status (e.g. livebirth or stillbirth).

ICD-10-AM procedural codes were used to identify interventions of interest, which included: elective caesarean delivery (16520–00; 16520–02), emergency caesarean delivery (16520–01; 16520–03), vacuum extraction (90469–00), forceps extraction (90468-00/01/02/03/04; 90470-02/04), medical and surgical induction of labour (90465-00/01/02/03/04/05), and episiotomy (90472–00). In the Irish context, caesarean deliveries coded as elective refer to a caesarean delivery which occurs prior to labour. Failed attempts at vacuum and forceps extraction (90469–01; 90468–05) which resulted in caesarean delivery were not included in the overall rates of operative vaginal delivery. Records with no indication of caesarean delivery or vacuum/forceps extraction were presumed to be non-operative vaginal deliveries. We also identified epidural/spinal anaesthesia usage (92506-sub-divisions; 92507-sub-divisions; 92508-sub-divisions; 92516–00), as this procedure is known to be associated with operative vaginal delivery.

From ICD-10-AM diagnostic codes, we identified potential confounders as maternal morbidities and delivery-related factors which are associated with increased risk of obstetric intervention. Given the wide spectrum of morbidities which may affect an individual pregnancy and childbirth, we decided *a priori* to focus on three primary conditions which are frequently diagnosed in pregnancy and are likely to impact on the decision for obstetrical intervention: heart disease, diabetes and placental disorders. These morbidities were clinician-defined according to local hospital policy. We classified heart disease as diagnosis of chronic rheumatic heart disease (I05-I09), ischemic heart disease (I25), pre-existing hypertensive diseases (I10-I13; I15; I27; O10), gestational-induced hypertensive diseases (O13; O14; O15), and cases of hypertension where onset was unspecified (O11; O16). Similarly, we created a composite category for diabetes, which included diagnosis of established diabetes (E10-14; O24.0,1,2,3), gestational-induced diabetes (O24.4), and cases where onset was unspecified (O24.9). Placental disorders included placental malformations (O43.1,2,8,9), placenta praevia (O44) and placental abruption (O45). Other common obstetric risk factors included previous caesarean delivery (O34.2; O75.7) and multiple birth (Z37.2,3,4,5,6,7).

For the descriptive analysis, the overall mode of delivery rate by health care coverage status was reported per 100 deliveries. We examined six-year trends in private health care coverage and mode of delivery by health care coverage status using Cochrane-Armitage tests for trend. Multivariate logistic regression analyses were conducted to examine the association between health care coverage status and (1) elective caesarean delivery; (2) emergency caesarean delivery; (3) operative vaginal delivery (vacuum, forceps extraction); (4) induction of labour (use of prostaglandins, oxytocin, artificial rupture of membrane and other medical/surgical interventions); and (5) episiotomy. Given the frequency of our outcomes of interest (>10%), adjusted odds ratios were corrected to estimate the corresponding relative risks [19]. Thus, throughout this manuscript, estimates are described in terms of risk rather than odds.

In the multivariate models, adjustment for confounders varied by the risk associated with the outcome of interest. For induction of labour [20], adjustments were made for age, heart disease, diabetes, placental disorders and previous caesarean delivery. For elective and emergency caesarean delivery, adjustments were made for age, heart disease, diabetes, placental disorders, previous caesarean delivery and multiple birth. In light of recent evidence, epidural anaesthesia [21] and induction of labour [22] were not considered as confounders for caesarean delivery. For operative vaginal deliveries, adjustments were made for age, heart disease, diabetes, previous caesarean delivery, multiple birth, induction of labour and epidural. The interaction term between induction of labour and epidural was also included in the model. Lastly, for episiotomy, adjustments were made for age, multiple birth and operative vaginal delivery.

Similar to previous work [23], in order to construct the most appropriate comparison groups for our outcomes of interest, consideration was given to the intrinsic hierarchy of delivery pathways. Since all pregnancies are theoretically “at risk” for elective caesarean delivery (*i.e.* a pre-labour caesarean delivery), in the first model, we compared the likelihood of elective caesarean delivery versus all other modes of delivery. However, only women who undertake a trial of labour are at risk for emergency caesarean delivery, operative vaginal delivery and induction of labour. Thus, in the subsequent models assessing these interventions, we excluded elective caesarean deliveries (n = 48,214). Specifically, the likelihood of emergency caesarean delivery was compared to vaginal deliveries (both operative and non-operative), and the likelihood of operative vaginal delivery was compared to emergency caesarean/non-operative vaginal delivery. Induction of labour was examined among women with an emergency caesarean or vaginal delivery. Lastly, only women with a vaginal delivery are at risk for episiotomy; therefore in our final model, we excluded both elective and emergency caesarean deliveries (n = 103,045), and compared the likelihood of episiotomy among women with an operative or non-operative vaginal delivery. Analyses were conducted using SAS V9.3 (SAS Institute Inc., Carey, NC, USA).

### Ethics statement

This study was exempt from institutional board review as hospital discharge records do not contain unique identifiers and are available for public access (University College Cork Clinical Research Ethics Committee, Ref. No. ECM4(g)05/08/08).

## Results

403,642 childbirth hospitalizations were reviewed. Over the six-year period, approximately one-third of maternities (30.2%) were booked privately. The percentage of private bookings steadily decreased over the study period, from 33.3% to 24.2% (results not shown; test for trend *p*-value < 0.001).

In the overall cohort, mode of delivery distribution differed by health care coverage status (Table 1). Women with private coverage were almost twice as likely to have an elective caesarean delivery (17.8% versus 9.4%). However, the emergency caesarean rate was similar between women with private and public coverage (14.3% versus 13.3% respectively), and only minor differences in the rates of operative vaginal delivery were observed.

**Table 1:** Distribution of maternal and obstetric characteristics for public and private deliveries, Ireland, 2005-2010

	**Private deliveries**	**Public deliveries**
	**(N = 122,072)**	**(N = 281,570)**
**Maternal characteristics**		
Age (years)		
<20	0.2 (286)	4.8 (13,585)
20 – 29	13.9 (17,006)	46.3 (130,292)
30 – 39	78.9 (96,298)	45.8 (128,831)
≥40	7.0 (8,482)	3.2 (8,862)
Morbidity		
Heart disease^a^	5.5 (6,732)	5.4 (15,251)
Diabetes mellitus^b^	1.4 (1,761)	2.3 (6,446)
Placental disorders^c^	1.2 (1,476)	1.1 (3,110)
**Mode of delivery**		
Caesarean, elective	17.8 (21,667)	9.4 (26,547)
Caesarean, emergency	14.3 (17,428)	13.3 (37,403)
Vaginal, operative, total^d^	18.1 (22,069)	14.9 (41,995)
Vacuum	13.2 (16,076)	11.5 (32,302)
Forceps	4.7 (5,734)	3.3 (9,256)
Combined	0.2 (259)	0.2 (437)
Vaginal, non-operative	49.9 (60,908)	62.4 (175,625)
**Induction of labour**^**e**^		
Prostaglandin	7.0 (6,986)	6.0 (15,273)
Oxytocin	3.7 (3,723)	3.5 (8,901)
Artificial rupture of membrane	6.1 (6,091)	4.7 (12,054)
Other^f^	16.4 (16,457)	12.1 (30,747)
Total induction	32.5 (32,622)	26.0 (66,320)
**Select procedures**		
Episiotomy^g^	29.5 (24,500)	20.9 (45,575)
Epidural usage^e^	64.1 (64,386)	50.4 (128,516)
**Other obstetric conditions**		
Previous caesarean delivery	15.1 (18,487)	8.7 (24,454)
Multiple birth	2.2 (2,693)	1.4 (4,039)

Note: Rates are % (N). Rates are based on all deliveries (n = 403,642) unless otherwise stated.

^a^Includes chronic rheumatic heart disease, ischemic heart disease and pre-existing/gestational-induced hypertensive diseases.

^b^Includes pre-existing and gestational-induced diabetes.

^c^Includes placental malformations, placenta praevia and placental abruption.

^d^Categories are not mutually exclusive.

^e^Rates are based on emergency caesarean and vaginal (operative and non-operative) deliveries only (n = 355,428).

^f^Includes medical and surgical methods (i.e. Bougie, cervical dilation and Foley’s catheter) not elsewhere specified.

^g^Rates are based on vaginal deliveries only (n = 300,597).

Nonetheless, there were underlying differences in case-mix between women with private and public coverage. Whereas more than three-quarters of women with private coverage were between 30 and 39 years old (78.9%), less than half of women with public coverage (45.8%) fell within this age bracket. No major differences in rates of heart disease or placental disorders were observed; though, reported rates of diabetes (established or gestational-induced) were modestly lower among women with private coverage. Among women with private coverage who undertook a trial of labour, all forms of induction of labour were higher, as was use of epidural anaesthesia. Furthermore, episiotomy rates for vaginal deliveries were substantially higher among women with private coverage. The rate of previous caesarean delivery among women with private coverage was nearly double that observed among women with public coverage; multiple gestations were also more frequent in women with private coverage.

**Figure 1:**
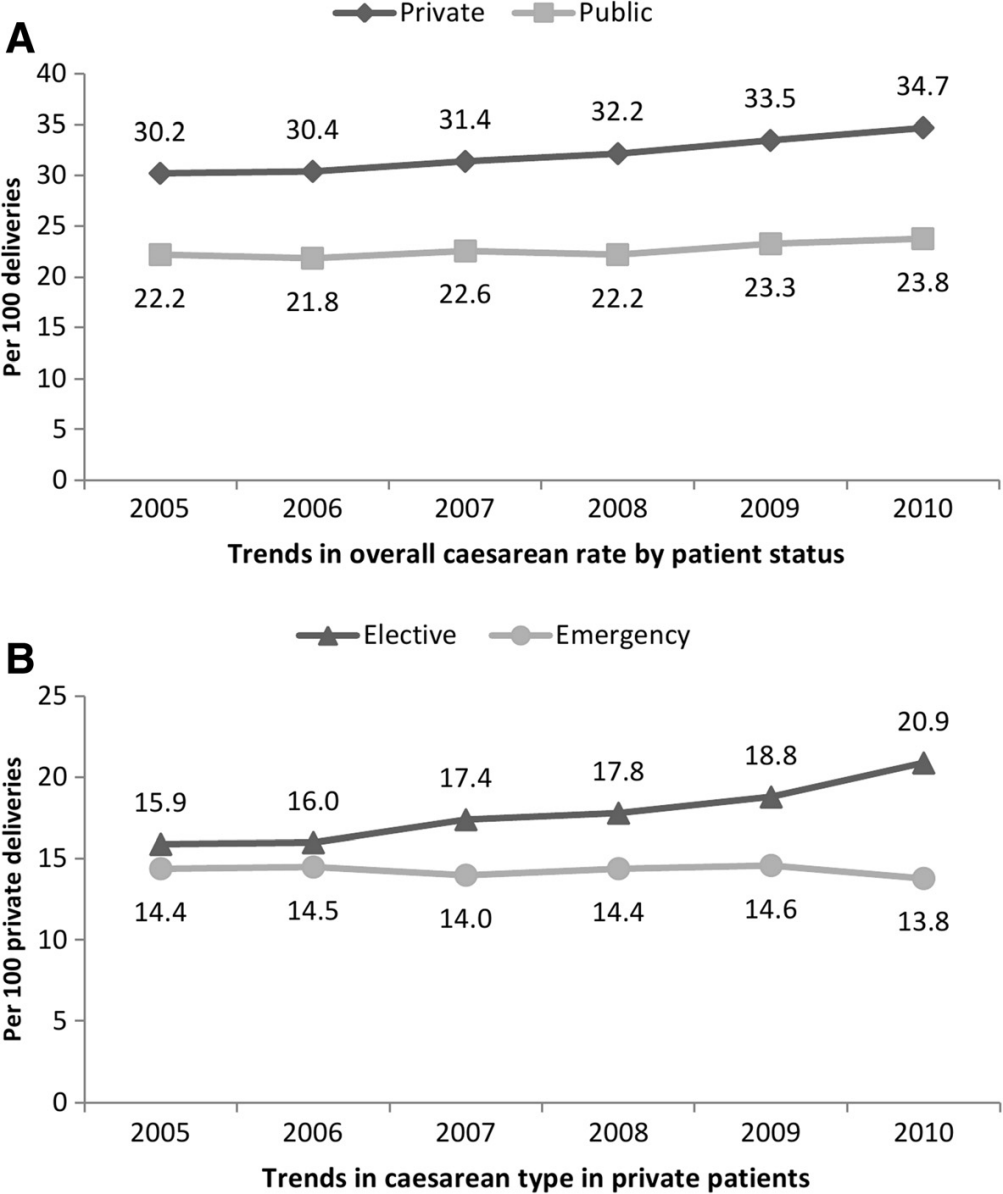
Trends in caesarean delivery by health care coverage status, Ireland, 2005-2010 (Panels A and B).

**Figure 2:**
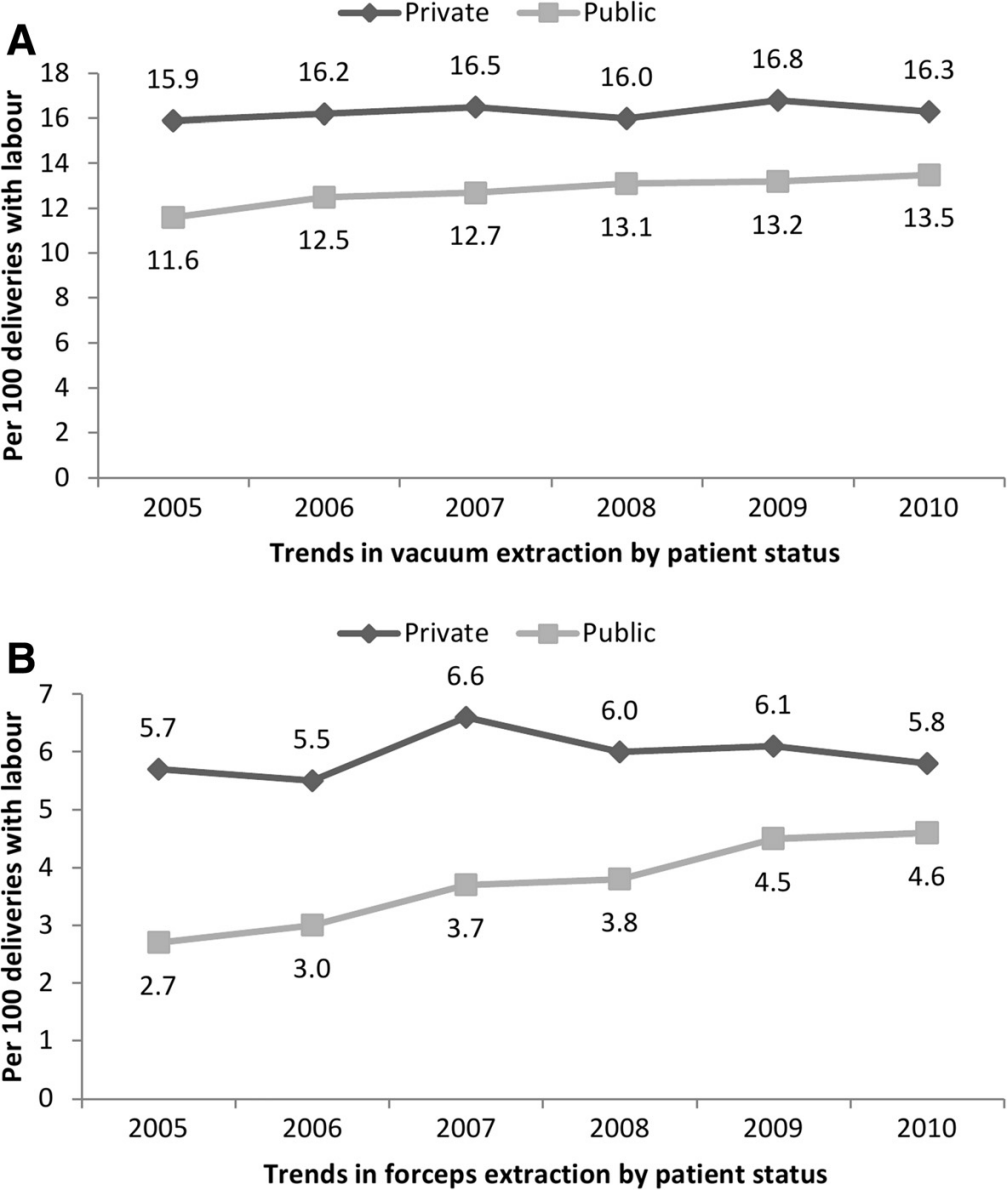
Trends in operative vaginal delivery by health care coverage status, Ireland, 2005-2010 (Panels A and B).

Changing patterns in obstetric intervention were evident between 2005 and 2010. Increasing caesarean delivery rates were more pronounced among women with private coverage (30.2% to 34.7%) than women with public coverage (22.2% to 23.8%) (test for trend p-value <0.0001; Figure 1, Panel A). The increasing caesarean rate in women with private coverage was predominately driven by an increase in elective caesarean deliveries (Figure 1, Panel B). Notably, there was a concurrent decrease in attempted trial of labour after previous caesarean (results not shown). Whereas rates of vacuum (test for trend p-value = 0.14) and forceps extraction (test for trend p-value =0.32) were stable among women with private coverage who underwent labour, significant increasing trends in operative vaginal delivery were observed among women with public coverage (test for trend p-values <0.0001; Figure 2, Panels A and B). Induction of labour rates were relatively stable in both groups of women (results not shown). In contrast, among women with a vaginal delivery, episiotomy rates significantly increased for both women with private (28.9% to 31.3%) and public coverage (19.0% to 22.7%) (results not shown, test for trend p-value <0.0001).

In the univariate models, women with private coverage had increased risks of caesarean delivery, operative vaginal delivery, induction of labour, and episiotomy; adjustment in the multivariate models did not attenuate observed risks (Table 2). After controlling for relevant obstetric risk factors, women with private coverage were 48% more likely to have an elective caesarean delivery, 13% more like to have an emergency caesarean delivery and 25% more likely to have an operative vaginal delivery. Risk of forceps extraction was higher than risk of vacuum extraction. Risk of induction of labour varied by type; however overall, women with private coverage were 27% more likely to have an induction of labour relative to women with public coverage. Compared to women with public coverage with a vaginal delivery, women with private coverage were 40% more likely to have an episiotomy.

**Table 2:** Unadjusted and adjusted relative risks for obstetric intervention in women with private versus public health care coverage, Ireland, 2005–2010

	**Unadjusted**	**Adjusted**
	**RR (95% CI)**	**RR (95% CI)**
**Induction of labour**^**a**^		
Total induction	1.25 (1.24–1.26)	1.27 (1.26–1.29)
Prostaglandin	1.16 (1.13–1.19)	1.18 (1.14–1.20)
Oxytocin	1.06 (1.02–1.10)	1.10 (1.05–1.13)
Artificial rupture of membrane	1.28 (1.24–1.32)	1.25 (1.22–1.29)
Other^b^	1.36 (1.34–1.38)	1.41 (1.38–1.44)
**Mode of delivery**		
Elective caesarean^c,d^	1.88 (1.85–1.91)	1.48 (1.45–1.51)
Emergency caesarean^d^	1.18 (1.16–1.20)	1.13 (1.12–1.16)
Vaginal, operative, total^e^	1.33 (1.32–1.35)	1.25 (1.22–1.27)
Vacuum^e^	1.27 (1.25–1.29)	1.19 (1.17–1.22)
Forceps^e^	1.57 (1.52–1.62)	1.39 (1.34–1.45)
**Select procedures**		
Episiotomy^f^	1.41 (1.39–1.43)	1.40 (1.38–1.43)

Note: All risk estimates are based on emergency caesarean or vaginal (operative and non-operative) deliveries (n = 355,428) unless otherwise stated.

^a^Adjusted for age, heart disease, diabetes, placental disorders, previous caesarean delivery.

^b^Includes medical and surgical methods not elsewhere specified.

^c^Based on all deliveries (n = 403,642).

^d^Adjusted for age, heart disease, diabetes, placental disorders, previous caesarean delivery and multiple birth.

^e^Adjusted for age, heart disease, diabetes, previous caesarean delivery, multiple birth, induction of labour and epidural. The interaction term between induction of labour and epidural was also included in the model.

^f^Based on vaginal deliveries only (N = 300,597); adjusted for age, multiple birth and operative vaginal delivery.

## Discussion

Irrespective of obstetric risk factors, we found that women who opted for private maternity care in Ireland were significantly more likely to have an obstetric intervention than women who opted for public care. Although such disparities have not been found in all settings [24,25], our research contributes to a growing body of evidence which supports such an association [1-11]. Due to fundamental differences between study designs and operational definitions, risk estimates describing the impact of private health care coverage on obstetric intervention are not directly comparable. However, assessing the influence of health care coverage status in a variety of health care settings is critical given that rates of obstetric intervention are likely impacted by a country’s prevailing model of obstetric care (i.e. midwife-led, obstetrician-led or shared care models) and health care system (i.e. socialised medicine or fee-for-service).

The variation in the overall caesarean rate between women with public and private coverage is of concern, particularly given that increasing trends in elective caesarean delivery were disproportionately higher among women with private coverage. Although generally a safe procedure, caesarean delivery is a major abdominal surgery associated with increased postpartum recovery time, higher risk of uterine complications in future pregnancies, and increased risk of respiratory morbidities for the infant [26]. While undoubtedly such trends are impacted by differences in obstetric profiles, our study suggests that health care coverage status is likely an independent risk factor for caesarean delivery.

Yet, health care coverage status is part of a broad spectrum of non-clinical reasons, including obstetrician preference [27,28], litigation fears [29-31], maternal preference [32,33], and fewer women attempting a trial of labour after previous caesarean [34,35]. For this reason, to better understand both clinical and non-clinical dynamics, in future studies of health care coverage status and caesarean delivery, mixed-method research would be a clear advantage. Given the heterogeneity in obstetric risk factors among women who receive a caesarean delivery, the quantitative component of such an analysis may consider performing a more detailed and standardised classification of caesarean delivery, for example by applying the Robson 10-Group Criteria [36]. The Robson 10-Group Criteria classify caesarean deliveries according to fetal position, number of gestations, parity, course of labour and gestational age, and thus this classification system inherently captures many important risk factors for obstetric intervention. Group-specific caesarean delivery rates can then be derived and compared between women at high and low risk for caesarean delivery. For example, whereas as higher rates of caesarean delivery can be expected among nulliparous breech pregnancies (Robson Group 6), lower rates would be expected among nulliparous, single cephalic, term pregnancies with spontaneous labour (Robson Group 1). Using this framework, qualitative research can be undertaken to investigate the different dynamics in different subgroups which present with varying levels of risk for caesarean delivery. Such research would ideally focus on clinical decision-making and the influence of the personal preferences of women versus maternity care professionals. We believe that this research design could greatly enhance our current understanding of the influence of private health care coverage on caesarean delivery.

Over the study period, we found a significant increase in the proportion of women with public coverage having an operative vaginal delivery; though interestingly, such trends were not observed among women with private coverage. In particular, the increase in forceps deliveries among women with public coverage was unexpected. Although increases in vacuum extraction have been reported in other settings, typically, this is coupled with a decrease in forceps extraction [5,37]. The increases we observed in operative vaginal delivery could potentially represent a more interventional practice style, or in contrast, a pro-active effort to avoid caesarean delivery. Similarly to caesarean delivery, operative vaginal delivery in and of itself is not without risk of increased maternal morbidity [38], and thus detailed clinical audit is warranted to further investigate observed patterns in women with public health care coverage.

We found that rates of episiotomy were significantly higher among women with private coverage, which is disconcerting given that liberal use of episiotomy may increase risk of severe perineal trauma, posterior perineal trauma, more extensive suturing and complications in healing [39]. In light of such evidence, major obstetrical societies have advocated restrictive use of episiotomy to decrease maternal morbidity [40,41]. Such guidelines have subsequently led to a decrease in rates over the past three decades [42,43]. We are unable to confirm why differences in episiotomy rates were observed in this population. Speculatively, however, uncomplicated deliveries in the public scheme are largely attended by midwives, who may be less likely to carry out an episiotomy [44].

Our study is subject to several biases. Firstly, residual confounding is of concern as we were not able to adjust for all maternal (e.g. parity, obesity, assisted conception, ethnicity and socio-economic status) and fetal (e.g. position, intrauterine growth restriction, macrosomia, heart rate) risks factors which may have increased risk of obstetric intervention. Given that HIPE is a minimal dataset with the primary objective of auditing overall clinical activity, these factors were not built into the original HIPE dataset. Parity in particular is a well-established risk factor for obstetric intervention, and previous reports from Ireland have shown that multiparous women are substantially less likely to have an operative vaginal delivery and slightly less likely to have a caesarean delivery [18]. If the distribution of parity (as well as other aforementioned factors unavailable in the HIPE dataset) differed by health care coverage status, estimates reported in this analysis may be biased. It is difficult, however, to speculate the directionality of such bias because certain risks may be higher in women with private coverage whereas other risks may be higher in women with public coverage. Notably, new variables continue to be introduced into the HIPE dataset, and commencing in 2012, information on parity will be available. However, to the authors’ knowledge, there are no published studies to date that report nationally representative figures of parity by health care coverage status. Linking hospital discharge data with maternal/infant medical charts and birth registration forms is another mechanism to counter this limitation, though linked datasets are currently not in compliance with current health information regulations in Ireland.

Secondly, reporting bias is an issue when using secondary administrative datasets, such as the HIPE database. Data extracted from hospital records may underreport the true extent of covariates and outcomes of interest in this population. Nonetheless, validation studies performed in other countries have found that hospital discharge datasets [45], including those coded using the ICD-10-AM [46], are highly sensitive and specific for many obstetric conditions and interventions. These findings, coupled with the previous findings which have demonstrated the accuracy and reliability of the HIPE database [16,17], lend to the credibility of our results.

Thirdly, the HIPE database lacks unique maternal identifiers, and therefore we were unable to link deliveries for women who had multiple pregnancies over the study period. Such information would have facilitated a useful sub-analysis focusing on women who alternated between private and public care in different pregnancies or to examine clustering by the unit of the individual woman. Lacking a unique identifier further impacted the use of a multi-diagnostic/procedural definition to extract childbirth hospitalisations [47], as such a metric would have increased the risk of duplicate records (*e.g.* in the case of transfers) [48]. Moreover, since the type of caesarean delivery (elective versus emergency) is not independently recorded in the HIPE database, this method would have also prevented the examination of caesarean type by health care coverage status, which we deemed highly important in this analysis. Notably, the proportion of childbirth hospitalisations missed using an outcome of delivery code is relatively small [47], and previous reports from Ireland have shown a high degree of concordance between estimates based on birth records versus outcome of delivery codes from hospital discharge records [18,49].

Lastly, we were unable to definitely discern if observed differences were a result of health care coverage status or the attending maternity care provider (*i.e.* obstetrician versus midwife). This is an important distinction that warrants further investigation. In our opinion, however, disparities in obstetric intervention are likely not attributed to solely one factor, but rather a confluence of factors. For this reason, we firmly advocate research which integrates the preferences of women and maternity care providers with clinical indications for intervention.

## Conclusions

In conclusion, our review of recent data from Ireland demonstrates that significant differences in obstetric intervention in women with private and public health care coverage persist and are unlikely to be explained by differences in clinical risk factors alone. While there is clear value in decreasing unnecessary obstetric intervention, how to best achieve this aim will likely require reassessment of obstetric management at both the local and national level. The incorporation of targeted strategies, such as timely implementation of evidence-based guidelines, mandatory secondary opinion and detailed consultations may be of benefit [50]. Successful initiatives would likely require a multi-dimensional approach which addresses concerns held by both the mother and health care provider.

### Details of ethical approval

This study was exempt from University College Cork Clinical Research Ethics Committee review as it used publicly available, anonymised data (Ref. No. ECM4(g)05/08/08).

## Competing interests

The authors declare that they have no competing interests.

## Authors’ contributions

JEL, DD and MM contributed to the design and analysis of the study. JEL and MM contributed to the drafting of the initial manuscript. JEL, DD, MM, SM and RAG contributed critical revision of the manuscript for intellectual content and approved its submission for publication. All authors read and approved the final manuscript.

## Pre-publication history

The pre-publication history for this paper can be accessed here:

http://www.biomedcentral.com/1471-2393/14/13/prepub
